# Developing and testing a framework to measure and monitor safety in healthcare

**DOI:** 10.1177/1356262214535735

**Published:** 2014-05

**Authors:** John Illingworth

**Affiliations:** Health Foundation, London, UK

**Keywords:** Communicating risk, risk management, medical disasters, medical regulation, public inquiries

## Abstract

The NHS excels at measuring incidences of past harm – whether it is falls or hospital-acquired infections – but research undertaken by Charles Vincent, Jane Carthey and Susan Burnett for the Health Foundation suggests past harm is only one element of what is needed to understand how safe care is. The researchers developed a framework to incorporate other necessary elements, such as anticipating and preparing for risks before they lead to harm to patients. In 2013, the Health Foundation road-tested this framework with staff in three NHS organisations and held a two-day summit with leaders from across the healthcare system to get feedback on its potential. This article presents the findings of this phase of work and sets it in the context of recent changes in the policy and regulatory landscape for patient safety in England. It concludes that the framework offers a great deal of potential for supporting organisations to understand the safety of their services. The framework could be most effective when used to identify the relative strengths and weaknesses of current safety measures, and when staff are given sufficient time, resource and support to consider the complex issues surfaced by the questions in the framework. This needs to be matched by a system of regulation which is aligned and mature, and an approach from NHS Trust Boards which welcomes information about the risks of its services.

## Introduction

The prevailing approach to understanding and improving patient safety in the NHS focuses on past failures, rather than anticipating and mitigating the risks facing patients, organisations and systems now and in the future. Over the past 15 years, we have witnessed appalling breaches of safety, most recently in the failings of care at Mid Staffordshire NHS Foundation Trust.^1^ Lurching from one crisis to the next, necessary but time-consuming inquiries have been undertaken to learn the lessons necessary to prevent their reoccurrence. To achieve ‘a system devoted to continual learning and improvement,’^2^ it is of course critical to understand the events and conditions which contribute to such catastrophes; but will this inquiry-led model of safety improvement, with the resulting stream of nationally-driven interventions, be sufficient to take us to the next stage of safety in healthcare?

**Figure 1.**
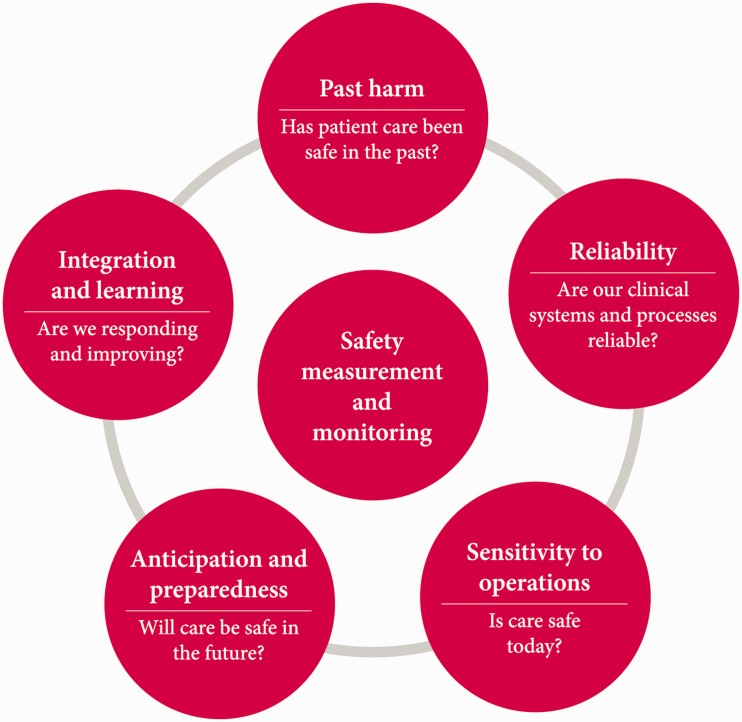
A framework for measuring and monitoring safety.

This retrospective mind-set has had a pervasive influence on how NHS organisations seek to, and are required to, measure and monitor the safety of their services. The NHS excels at measuring incidences of past harm – whether it is falls or hospital acquired infections^3^ – and this approach has delivered some considerable success in tackling specific factors that contribute to patient harm. However, research undertaken by Vincent et al.^4^ for the Health Foundation suggests past harm is only one part of what is needed to understand how safe care is. The researchers identified other necessary elements, which can be more challenging to understand, such as anticipating and preparing for risks *before* they lead to harm to patients. The researchers brought together the different elements in a framework (Figure 1). Based on the research, the Health Foundation developed some prompts to support staff in the NHS wishing to apply the framework to their own organisations (Table 1).

**Table 1.** Prompts for NHS staff to apply the framework developed by Vincent et al.^4^ to better understand the safety of care.

1. *Past harm – has patient care been safe in the past? • *Identify the different types of harm that can exist in your setting *• *Use a range of safety measures, while understanding their strengths and limitations *• *Ensure the measures are valid, reliable and specific 2 *Reliability – are our clinical systems and processes reliable? • *Specify the level of reliability you would expect in areas of standardised practice *• *Use local and national audits and initiatives to monitor reliability *• *Understand what contributes to poor reliability 3. *Sensitivity to operations – is care safe today? • *Select an appropriate mix of formal and informal safety monitoring mechanisms *• *Use the information to take action to avert safety issues *• *Reflect on whether current structures and committees enable timely action to be taken 4. *Anticipation and preparedness – will care be safe in the future? • *Don’t wait for things to go wrong before trying to improve safety *• *Explore new opportunities to develop systematic ways to anticipate future risks *• *Use a variety of tools and techniques to build an understanding of the factors that give rise to safety issues 5. *Integration and learning – are we responding and improving? • *Use the analysis of incidents to reveal the wider issues in the system *• *Place more emphasis on learning, feedback and action than simply on data collection *• *Integrate and tailor information to make it meaningful from the ward to the board

In 2013, the Health Foundation road-tested this framework with staff across three NHS organisations, and held a two-day summit with leaders in the healthcare system to get feedback on its potential. This article presents the findings of this phase of work, and sets it in the context of recent changes in the policy and regulatory landscape for patient safety in England. It concludes that the framework offers a great deal of potential in supporting organisations to understand the safety of their services. The framework could be most effective when used to identify the relative strengths and weaknesses of current safety measures, and when staff are given sufficient time, resource and support to consider the complex issues that the questions in the framework surface. There was general agreement from healthcare leaders that a move to understanding and measuring factors that relate to future risk was critical to improving safety. However, this needs to be matched by an approach from regulators that is aligned and mature, rather than overlapping and prescriptive. This article concludes that the public, front line health professionals, managers and leaders alike should be meaningfully engaged in an open conversation about how to build a safer NHS.

## New directions in patient safety policy

The publication of the Healthcare Commission’s investigation into seemingly high mortality rates in patients admitted as emergencies at Mid Staffordshire NHS Foundation Trust in 2009^5^ led to a series of government interventions which continue to reshape the landscape for patient safety policy in England. These interventions include a range of initiatives designed to improve the measurement and monitoring of patient safety, announced in the government’s initial^6^ and final^7^ responses to the Mid Staffordshire Public Inquiry.^8^ For instance, NHS England will develop a dedicated public hospital safety website to draw together up-to-date information on patient safety factors. They will encourage further the use of the NHS Safety Thermometer^9^ – a local improvement tool for measuring, monitoring and analysing patient harms and harm-free care – and trusts will be required to publish their ward-level staffing figures on a monthly basis.

On the regulation of healthcare, the Care Quality Commission (CQC) – the independent regulator of health and adult social care in England – is making significant changes to how it will monitor safety, including the enhanced use of surveillance information to determine what, where and when it inspects, and the introduction of a new system of ratings of the quality and safety of provider organisations.^10^ The NHS Litigation Authority has revised its methodology for calculating contributions to the clinical negligence scheme for trusts (CNST), ensuring that organisations with fewer, less costly claims pay less into the scheme.^11^

In 2013, NHS England, led by its Medical Director Sir Bruce Keogh, investigated 14 hospital trusts that were persistent outliers on mortality data.^12^ Several issues were identified ‘that had to be tackled immediately in order to avoid causing possible harm to patients.’ Around the same time, children’s heart surgery at Leeds General Infirmary was suspended for two weeks pending the investigation of data which showed unusually high mortality rates. The review concluded that the unit ‘does not have an excessive mortality.’^13^ These developments emphasise the critical role that data will play in monitoring the safety of healthcare at the macro level, with information used as a warning system to trigger further investigation to determine what the information means.

Much of the data being used for these purposes still relate to the past experiences of patients. For example, the NHS Litigation Authority’s calculation of CNST contributions will be based on previous compensation claims. The surveillance information used by the CQC will include the monitoring of mortality data, but it would be inappropriate to think of an excess number of deaths as a ‘smoke detector’ to poor care.^14^ Such information is valuable when used as a trigger for further inquiry and to analyse changes in performance over time (if the data are collected consistently and accurately). However, while it provides a picture of the extent to which patients have been harmed in the past, this type of data alone does not tell us how safe a system is, how resilient it would be to unexpected events or the extent to which patients will be safe using services today or tomorrow.

As announced in *Hard Truths*, NHS trusts will now collect and publish ward-level staffing data.^7^ This is a welcome development. Staff numbers (including consideration of skill levels and patient acuity) are one of several key factors that affect the ability of a unit to deliver safe care and therefore it is positive to insist on having reliable real-time data. However, this data will only be effective when it is used to inform operational decisions on a frequent, probably shift-by-shift, basis. It remains to be seen if requiring trusts to do this on a monthly basis, as a central mandate, will achieve the result intended.

Asked by the Prime Minister to help the NHS achieve ‘zero harm,’ Don Berwick’s National Advisory Group on the Safety of Patients in England concluded that ‘patient safety cannot be improved without active interrogation of information that is generated primarily for learning, not punishment, and is for use primarily at the front line.’^2^ Similarly, Vincent and colleagues^4^ recommended that there ought to be ‘a blend of externally required metrics and local development.’ This balance between nationally imposed initiatives and locally-determined goals lies at the heart of much of the debate around the response to events at Mid Staffordshire. The framework for measuring and monitoring safety (Figure 1) seeks to create a shared philosophy at different levels of the system, but its main value will lie in its use at the front line of care.

## A framework for measuring and monitoring safety

In *The measurement and monitoring of safety*,^15^ Charles Vincent and colleagues brought together evidence from the published literature as well as a survey of current practice. They shifted the emphasis away from focusing solely on past cases of harm and more towards real-time performance, and measures that relate to future risks and the resilience of organisations. The framework they developed suggests five dimensions and questions that can be used by individuals, units, teams, departments and organisations across all healthcare settings – primary, community, mental health and acute care. These dimensions and questions are described below, along with feedback from a range of staff groups when the framework was road-tested in three NHS organisations.

1. *Past harm – has patient care been safe in the past?* In all three organisations, this was the question that staff at all levels – clinicians, managers and board members – felt most comfortable in answering, using the types of information that would be familiar to people working in the NHS, such as complaints data and infections rates. However, the research suggests that the multiple types of harm that can occur (e.g. harm from treatment or delayed or inadequate diagnosis) require a broad range of measures to give the strongest understanding of past harm. This includes mortality statistics, systematic record review, case note review and incident report analysis.
2. *Reliability – are our clinical systems and processes reliable?* Process reliability was an area familiar to many of the staff who tested the framework, but there was recognition that measuring and creating true system reliability is complex. The research states that reliability is meaningful where care has a high degree of standardisation (e.g. managing acute asthma in emergency departments), and audits can be undertaken to monitor compliance. However, it is important to be wary and ensure that ‘ticking a checklist’ does not provide false reassurance about the quality and safety of care.
3. *Sensitivity to operations – is care safe today?* Staff at all three organisations highlighted the importance of the ‘softer’ signals on the ground – such as the demeanour of the ward manager, the ‘chatter’ of staff and the physical state of the ward – as well as ‘harder’ information such as staffing levels and patient acuity. The research describes a state of ‘heightened awareness’ that enables action to be taken to tackle identified problems before they threaten patient safety.
4. *Anticipation and preparedness – will care be safe in the future?* The use of information and approaches to identify future hazards was the area that staff had the most difficulty in assigning current measures, reflecting the findings of the research. While there are some more sophisticated tools, such as Human Reliability Analysis, that help to indentify specific risks, safety culture assessments, sickness absence and staffing rates can help to forecast organisations’ resilience to unexpected events.
5. *Integration and learning – are we responding and improving?* The way that information is integrated and acted upon was a particular focus for the managers in the three organisations, particularly the challenges of being able to translate data so that it is meaningful and to use it to take action, given the time lags in data being made available to them. The research underlined the importance of drawing together disparate pieces of information to spread the learning right across organisations.

Overall, the feedback from the trusts testing the framework was very positive, and demonstrated that it can be applied in a variety of ways to improve the understanding of safety – as a diagnostic or ‘gap analysis’ tool, as a discussion vehicle or as a template around which to build a broad range of safety measures. The framework enabled people to think about stopping the collection of some measures that were not adding value, as well as suggesting new ones. It worked most effectively as part of an honest assessment about where teams’ strengths and weaknesses are, and when the questions are considered deeply rather than ‘ticking them off’. Individuals and organisations should be encouraged to look beyond the ‘deceptively simple’ framework diagram, and use the detail and case studies in the research report to really examine what it might mean in each clinical context.

In conclusion, this framework calls for action across different levels within NHS organisations:

- *Front line health and care professionals* should reflect on the value of data currently collected, and identify opportunities to collect more meaningful data about the safety of their services.
- *Managers and support staff* should work with clinical staff to ensure that learning, feedback and action is prioritised following the review of safety information.
- *Board members* should seek information which identifies where the gaps are in their organisation’s safety measurement, rather than seek reassurance about the way things are currently done.

## Time for an open conversation about safety

In *The measurement and monitoring of safety*,^15^ the researchers set out how organisations, and the teams within them, can seek out a range of measures across different dimensions to better understand how safe their services are. However, the context that NHS organisations are operating in has a significant effect on their ability to do this in a constructive way. The shift in thinking that is proposed to take safety improvement to the next level is to proactively root out the factors that contribute to unsafe care, and to identify risks in the system before they lead to harm to patients. It remains to be seen if NHS boards are willing to hear the difficult messages about the potential risks in their services, and whether the regulators are ready to support, rather than penalise, an organisation that has identified these risks even when they are proactively managing them.

The recent experience at Kettering General Hospital NHS Foundation Trust illustrates both the opportunities and challenges facing the way we measure and monitor safety, and the reticence that remains about reactions to such difficult messages. Following the tragic death of a woman after an appendix operation, the trust identified 43 ‘care delivery problems,’ of which six had a direct impact on the death of the patient.^16^ This was reported by the trust following a request made under the Freedom of Information Act.^15^ The trust then decided not to make the findings of the investigation public, for fear of ‘endangering the mental health’ of staff.

If we are committed to introducing a duty of candour where staff are open and honest when something goes wrong, the next logical step is to promote a culture of candour about the risks associated with healthcare, and the efforts that are being made to mitigate those risks. In order for organisations to proactively mitigate risk they must feel that they can own these challenges more openly. This would be the sign of an NHS truly committed to building a safer system.

## Notes

1 The various investigations and inquiries culminated in the Report of the Mid Staffordshire NHS Foundation Trust Public Inquiry in February 2013.

2 NHS England, 2013. A promise to learn – a commitment to act: improving the safety of patients in England

3 The data for these harms is available from the Health and Social Care Information Centre through its publication of NHS Safety Thermometer reporting.

4 Vincent C, Burnett S and Carthey J. The measurement and monitoring of safety. The Health Foundation, 2013

5 Healthcare Commission, 2009. Investigation into Mid Staffordshire NHS Foundation Trust.

6 Department of Health, 2013. Patients First and Foremost: The Initial Government Response to the Report of the Mid Staffordshire NHS Foundation Trust Public Inquiry.

7 Department of Health, 2013. Hard Truths: The Journey to Putting Patients First.

8 Report of the Mid Staffordshire NHS Foundation Trust Public Inquiry in February 2013.

9 Taken from the Health and Social Care Information Centre.

10 Care Quality Commission, 2013. A new start. Consultation on changes to the way CQC regulates, inspects and monitors care.

11 NHS Litigation Authority. Safety and Learning Service, Questions and Answers.

12 Professor Sir Bruce Keogh, 2013. Review into the quality of care and treatment provided by 14 hospital trusts in England: overview report

13 NHS England, 2014. Leeds Children’s Heart Surgery Services Review.

14 Care Quality Commission, 2013. A new start. Consultation on changes to the way CQC regulates, inspects and monitors care.

15 Ibid.

16 Reported in BBC News article ‘Kettering Hospital withholds death inquiry findings’, 21 January 2014.

